# The influence of interdependence and a transparent or explainable communication style on human-robot teamwork

**DOI:** 10.3389/frobt.2022.993997

**Published:** 2022-09-08

**Authors:** Ruben S. Verhagen, Mark A. Neerincx, Myrthe L. Tielman

**Affiliations:** ^1^ Interactive Intelligence, Intelligent Systems Department, Delft University of Technology, Delft, Netherlands; ^2^ Human-Machine Teaming, Netherlands Organization for Applied Scientific Research (TNO), Amsterdam, Netherlands

**Keywords:** human-robot teamwork, explainable AI, transparency, explainability, interdependence, user study, human-agent teaming, communication

## Abstract

Humans and robots are increasingly working together in human-robot teams. Teamwork requires communication, especially when interdependence between team members is high. In previous work, we identified a conceptual difference between sharing what you are doing (i.e., being transparent) and why you are doing it (i.e., being explainable). Although the second might sound better, it is important to avoid information overload. Therefore, an online experiment (*n* = 72) was conducted to study the effect of communication style of a robot (silent, transparent, explainable, or adaptive based on time pressure and relevancy) on human-robot teamwork. We examined the effects of these communication styles on trust in the robot, workload during the task, situation awareness, reliance on the robot, human contribution during the task, human communication frequency, and team performance. Moreover, we included two levels of interdependence between human and robot (high vs. low), since mutual dependency might influence which communication style is best. Participants collaborated with a virtual robot during two simulated search and rescue tasks varying in their level of interdependence. Results confirm that in general robot communication results in more trust in and understanding of the robot, while showing no evidence of a higher workload when the robot communicates or adds explanations to being transparent. Providing explanations, however, did result in more reliance on RescueBot. Furthermore, compared to being silent, only being explainable results a higher situation awareness when interdependence is high. Results further show that being highly interdependent decreases trust, reliance, and team performance while increasing workload and situation awareness. High interdependence also increases human communication if the robot is not silent, human rescue contribution if the robot does not provide explanations, and the strength of the positive association between situation awareness and team performance. From these results, we can conclude that robot communication is crucial for human-robot teamwork, and that important differences exist between being transparent, explainable, or adaptive. Our findings also highlight the fundamental importance of interdependence in studies on explainability in robots.

## 1 Introduction

Increasingly, humans and robots will be working together in human-agent/robot teams (HARTs). Robots often outperform humans with respect to rapid, rational, and repetitive decision-making, thanks to their processing speed and memory capacity (van Diggelen et al., 2019). On the other hand, humans are usually still better at handling uncertainty and unexpected situations. HARTs make use of this unique combination of abilities.

HARTs can perform tasks where human and robot hardly depend on each other and can execute their individual actions independently (Singh et al., 2017). However, HARTs can also engage in joint activities in which what the human does depends on what the robot does (and vice-versa) over a sustained sequence of actions (Johnson et al., 2014). In such joint activities, the human and robot are interdependent and effective coordination and collaboration become crucial (Johnson et al., 2014; Singh et al., 2017).

Several factors are crucial when human and robot are interdependent, such as mutual trust and understanding; shared mental models; observability, predictability, and directability; and transparency and explainability (Klein et al., 2004; Salas et al., 2005; Johnson et al., 2014; Johnson and Vera, 2019). Unfortunately, many of these factors are still lacking in contemporary HARTs. For example, robots often demonstrate poor transparency and explainability, making it hard for human teammates to properly understand their inner workings, behavior, and decision-making (Lipton, 2018; Anjomshoae et al., 2019; Miller, 2019). This, in turn, negatively affects factors like mutual trust and understanding, eventually resulting in decreased global team performance (Johnson et al., 2014; Johnson and Vera, 2019).

Explainable AI (XAI) research, methods, and techniques emerged as a means of making AI-systems more understandable to humans (Gunning, 2017). Unfortunately, the field of XAI is characterized by a plethora of related but often ill-defined and inter-changeably used concepts like transparency, interpretability, explainability, and understandability (Verhagen et al., 2021). We addressed this issue by proposing a framework that unambiguously defined and related these concepts in a coherent and concise manner (Verhagen et al., 2021). This framework makes a distinction between robot transparency and explainability as different communication styles, with the former referring to the disclosure of information and the latter to also clarifying disclosed information using explanations.

One of the main goals within XAI community and research is the development of personalized, context-dependent and adaptive robots (Anjomshoae et al., 2019; De Visser et al., 2020). So instead of implementing robots characterized by fixed transparency or explainability, developing robots able to adapt their communication according to context and intended user. However, to do this we need to first understand how different communication styles like transparency and explainability exactly influence teamwork in different interdependency conditions. So far, very little work has examined the influence of interdependence between human and robot on human-robot teamwork outcomes, let alone the interaction between communication style and interdependence (O’Neill et al., 2020).

Therefore, this exploratory study will investigate the effects of different robot communication styles on crucial HART factors like team performance, trust, workload, situation awareness (SA) of the robot, and understanding. We will examine these effects across two levels of interdependence between the human and robot (high vs. low). To do this, we conducted a user study in a simulated environment where human participants collaborated with a virtual robot during a search and rescue task. The remainder of the paper is structured as follows. In Section 2 we discuss the relevant literature related to our study. Next, in Section 3 we describe how we conducted the user study, followed by the results in Section 4. Finally, we present a discussion and conclude our work in Section 5.

## 2 Background & related work

### 2.1 Interdependence

Interdependence in a team can be due to the relationships between team members and the task to execute. Four types of task interdependence have been identified: pooled, sequential, reciprocal, and team (Singh et al., 2017). Pooled task interdependence concerns the execution of tasks independently and without any interaction, whereas in sequential task interdependence, tasks are performed in a sequential order and team members have to wait for previous team members to complete their task. Reciprocal task interdependence involves team members taking turns in completing part of the task, while in team task interdependence, team members execute their individual tasks concurrently and may execute joint actions (Johnson et al., 2014; Singh et al., 2017). These task interdependence types form a hierarchy representing increasing needs for coordination and levels of dependence between team members. However, these task interdependence types are unable to capture the nuances of close collaboration between humans and robots working jointly on a task (Johnson et al., 2014). To capture these nuances of close collaboration, interdependence relationships between team members are required.

Johnson et al. (2014) define these interdependence relationships as the set of complementary relationships that human and robot rely on to manage required (hard) and opportunistic (soft) dependencies in joint activity. Their definition highlights the importance of dependencies and joint activity in interdependence between humans and robots working as team members. Joint activity is closely related to team task interdependence and relates to situations in which what the human does depends on what the robot does (and vice-versa) over a sustained sequence of actions (Johnson et al., 2014). For example, when a human-robot team engages in an urban search and rescue task. Such joint activity gives rise to required (hard) and opportunistic (soft) dependencies/interdependence relationships between team members. Hard interdependence stems from a lack of capacity (e.g., knowledge, skills, abilities, and resources) required to competently perform an activity individually (Johnson et al., 2014). For example, an explore robot/drone during urban search and rescue lacking the capacity to transport victims. In contrast, soft interdependence is optional and opportunistic, arising from recognizing opportunities to be more effective and efficient by working jointly (Johnson et al., 2014).

These different types of task interdependence and interdependence relationships (and their combinations) can give rise to high and low interdependence scenarios for human-robot teams. For example, when a human-robot urban search and rescue team allocates the task of exploring the disaster site to the robot and executing rescue operations to the human, human and robot may hardly depend on each other and execute their individual actions independently without much interaction (i.e., low interdependence). In contrast, human-robot teams can also engage in joint activities and actions in which both parties are mutually dependent on each other and where the human might need to support the robot (and/or vice-versa) for specific activities (i.e., high interdependence). For example, the same team can also explore a collapsed building together where both parties need to know which team member assessed which room, or where in case a victim is detected by the robot, the human needs to provide support with assessing the victim’s health status. So far, little work has been conducted on the effects of varying interdependence levels between human and robot on human-robot teamwork outcomes, and even less on the interaction between robot communication styles like transparency and explainability and different interdependence levels.

### 2.2 Robot communication and human-agent/robot teamwork

Several studies did investigate the effects of XAI on relevant human-agent/robot teamwork (HART) factors like trust, workload, and operator performance (Mercado et al., 2016; Selkowitz et al., 2017; Wright et al., 2017; Chen et al., 2018). These studies largely agree that operator performance and trust in the XAI system increase when it shares more reasoning information, and without detriment to workload (Mercado et al., 2016; Selkowitz et al., 2017; Chen et al., 2018). None of the studies, however, investigated the effects of communication style on global team performance, or how interdependence between human and robot affects trust, workload, and performance. Moreover, in all studies the XAI system served as an assistant of the human participants rather than as an equal team member. Our study will fill these gaps by examining the effects of robot communication style on global team performance, in HARTs where the robot is an equal team member, and across different levels of interdependence.

Other works investigated the relationship between robot information sharing and team performance, using a testbed (Blocks Worlds for Teams) similar to the one used in our study (Harbers et al., 2012b,a; Li et al., 2016; Singh et al., 2017; Wei et al., 2014). The Blocks World for Teams (BW4T) task is to deliver a sequence of coloured blocks in a particular order while working together in a team. The task is executed in a virtual environment containing rooms in which blocks are hidden, and a drop zone where blocks can be delivered. These studies have reported mixed results across different conditions. For example, most of them investigated artificial agent teams rather than human-agent/robot teams (Harbers et al., 2012b; Wei et al., 2014; Li et al., 2016; Singh et al., 2017). In addition, almost all examined the influence of shared mental model components (goals vs. world knowledge) on performance, rather than providing more or less reasoning information (Harbers et al., 2012b; Wei et al., 2014; Li et al., 2016; Singh et al., 2017). For example, Li, Sun, and Miller showed that in a high interdependence scenario containing joint actions, sharing goals was more effective than when the agent shared both goals and world knowledge with the human (Li et al., 2016). Harbers et al. did examine the effects of agents explaining their behavior on human-agent/robot teamwork (Harbers et al., 2012a). Their results showed that explanations about agent behavior did not always lead to better team performance, but did impact user experience in a positive way.

None of the studies in the BW4T testbed examined human-agent/robot teams across different levels of interdependence between human and agent, a gap that our study will fill. Furthermore, most of the discussed studies examined the influence of shared mental components on team performance. In this study we are not interested in this distinction, since we believe both goals and world knowledge are crucial for carrying out the task most efficiently. Instead, we will investigate how different communication styles affect human-agent/robot teamwork across two levels of interdependence. These different communication styles give rise to more and less detailed mental models of the agent, rather than omitting crucial components like world knowledge or goals.

### 2.3 Robot transparency vs. explainability

In our previous work we proposed a framework that makes a distinction between the communication styles transparency and explainability (Verhagen et al., 2021). This framework addresses the lack of agreement regarding the definitions of and relations between the key XAI concepts of transparency, interpretability, explainability, and understandability. More specifically, the framework discriminates between robot interpretability and understandability as passive and subjective system characteristics concerning user knowledge of the robot. In contrast, we defined robot transparency and explainability as active and objective characteristics involved with disclosing and clarifying relevant information. Transparency was defined as the disclosure of relevant system elements to users (e.g., robot decisions or actions), enabling users to access, analyze, and exploit this disclosed information (i.e., interpret). In contrast, we defined explainability as the clarification of disclosed system elements by providing information about causality and establishing relations with other system elements. Ultimately, these definitions resulted in the classification of three types of robots: incomprehensible, interpretable, and understandable. We argued transparency can make incomprehensible robots interpretable, and explainability can make interpretable robots understandable.

The third type of communication style not included in this framework but under investigation in this study, is adaptive communication. Miller discussed several factors to consider for such adaptive communication, such as epistemic relevance with respect to the user’s mental model, or what has been explained already (Miller, 2019). In addition to these user factors, a relevant contextual factor to consider is time pressure. Time pressure can decrease thorough and systematic processing of information while increasing selectivity of information processing, reducing both performance and decision-making quality (Maule and Edland, 2002; Schreuder and Mioch, 2011). Therefore, reducing communication frequency of an agent when time pressure is high seems beneficial to HART performance and trust, for example by only communicating the most important information. Unfortunately, the current implementation and experimental investigation of adaptive robot communication is limited, even more in the context of human-robot teamwork (Anjomshoae et al., 2019; De Visser et al., 2020). Our implemented adaptive style adjusts its communication based on both relevancy and time pressure.

### 2.4 Evaluating robot communication in human-agent/robot teamwork

The discussed studies showed several ways of evaluating XAI efficiency in a HART context, such as operator - and team performance, trust in the XAI system/robot, and workload during the task. Despite these different metrics, there is still a need for new metrics to assess XAI efficiency, specifically objective ones (Anjomshoae et al., 2019; Sanneman and Shah, 2020). Sanneman and Shah proposed an objective metric for assessing XAI effectiveness: the (modified) Situation Awareness Global Assessment Technique to measure SA of the XAI system’s behavior processes and decisions (Endsley, 1988; Sanneman and Shah, 2020), which we will adopt. First, situational information needs related to AI behavior should be thoroughly defined according to a process like the Goal Directed Task Analysis (Endsley, 2017). Next, simulations of representative tasks should be frozen at randomly selected times, followed by evaluating user knowledge of these predefined informational needs. The answers to these questions can be compared against the ground truth state of the world, providing an objective measure of the user’s SA of the AI.

## 3 Methods

To test the effects of different robot communication styles on human-robot teamwork across different levels of interdependence between human and robot, an experiment was conducted. In this experiment, we aimed to investigate the effect of four different robot communication styles on trust, reliance, workload, situation awareness, team performance, human contribution, communication frequency, and system understanding. Moreover, we aimed to also study whether interdependence between human and robot had any influence on this effect.

### 3.1 Design

The experiment had a 2 × 4 mixed design with interdependence between human and robot as the within-subjects independent variable and robot communication style as the between-subjects independent variable. Interdependence consisted of two conditions (low and high), robot communication style of four conditions (silent, transparent, explainable, adaptive). During the low interdependence condition, participants hardly depended on their robot teammate (and vice versa) since the work was split between the two. In contrast, during the high interdependence condition the participants and robot were highly dependent on each other because we removed the work division and added hard interdependence relationships stemming from a lack of robot capacity to carry critically injured adults and distinguish between kids. Which interdependence condition the participants completed first was counterbalanced, resulting in two order conditions.

### 3.2 Participants

We recruited 72 participants from the different universities’ mailing lists and personal contacts (23 females, 48 males, and one preferred not to say). Fifteen participants had an age range of 18–24 years old, 56 participants of 25–34 years old, and one participant was between 55–64 years old. In terms of education, one participant did some high school without obtaining a diploma, two participants were high school graduates, two participants obtained some college credit but no degree, three participants obtained an associate degree, 15 participants obtained a Bachelor’s degree, 47 participants obtained a Master’s degree, and two participants obtained a PhD degree or higher. With respect to gaming experience, 27 participants played video games several times a year, 24 participants several times a month, 12 participants several times a week, and nine participants played video games on a daily basis. Each participant signed an informed consent form before participating in the study, which was approved by the ethics committee of our institution (ID 1676).

Since each participant teamed up with a robot characterized by one of the four communication styles and one of the two interdependence order conditions, it was important to control for age, gender, education, and gaming experience across the communication style and order conditions. For gender, we conducted a Chi-square test of homogeneity while for age, education, and gaming experience a Kruskal-Wallis test was conducted. Results showed no significant differences between communication style conditions for any of the demographic factors gender (*χ* ^2^ (6) = 7.29, *p* = 0.29), age (*χ* ^2^ (3) = 0.76, *p* = 0.86), education (*χ* ^2^ (3) = 0.34, *p* = 0.95), and gaming experience (*χ* ^2^ (3) = 0.31, *p* = 0.96), indicating that participants were evenly split over the communication style conditions. Moreover, results showed no significant differences between interdependence order conditions for gender (*χ* ^2^ (2) = 2.95, *p* = 0.23), age (*χ* ^2^ (1) = 0.75, *p* = 0.39), education (*χ* ^2^ (1) = 2.07, *p* = 0.15), and gaming experience (*χ* ^2^ (1) = 0.21, *p* = 0.65).

### 3.3 Hardware and software

To run this experiment we used a Dell laptop, a Virtual Machine (Ubuntu 20.04.2 LTS), and the Human-Agent Teaming Rapid Experimentation (MATRX: https://matrx-software.com/) software, a Python package specifically aimed at facilitating human-agent teaming research. The Dell laptop was used to access the Virtual Machine, from which a MATRX world was launched. This two-dimensional grid world contained and tracked the information needed to simulate the agents performing tasks in our environment.

### 3.4 Environment

**FIGURE 1:**
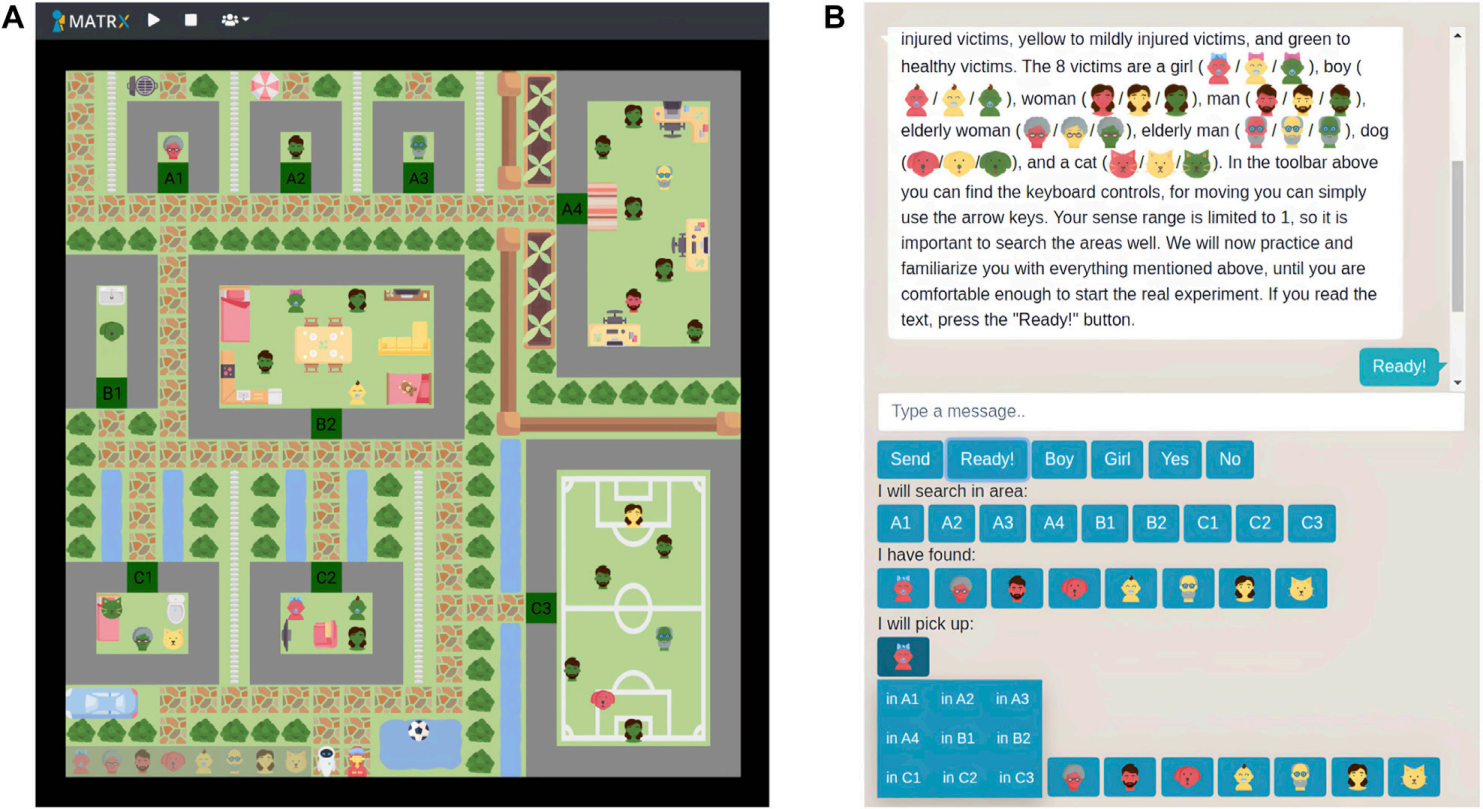
**(A)** God view of the MATRX world used for this study. The lower left corner of the world shows the drop zone with eight victims to search and rescue. Next to the drop zone are RescueBot and the human avatar at their starting positions. **(B)** the chat functionality and buttons used by participants to communicate. In addition, the different victim and injury types can be seen. Buttons existed for each area and goal victim to search and rescue.

To access the MATRX world and control their corresponding human agent, participants opened a link in either Chrome or Firefox. In contrast, the experimenter viewed the world with the so called God agent, making it possible to perceive everything and start, pause, and stop the world. We built a world consisting of nine areas, 28 collectable objects, and at least one drop zone (Figure 1A). Furthermore, we added an autonomous virtual robot and human agent to our world, and designed an environment in which these two agents had to collaborate during a search and rescue task. Two different worlds were created, one for each interdependence condition, varying with respect to the drop zone(s) and victim distribution. The low interdependence world consisted of two drop zones with four victims each, whereas the high interdependence world contained just one drop zone with eight victims.

We created the following eight victim types making up the world’s collection goal: boy, girl, man, woman, elderly man, elderly woman, dog, and cat. In addition, we created the following injury types: critical, mild, and healthy. Injury type was represented by the color of the victims, where red reflected critically injured, yellow mildly injured, and green healthy victims (see Figure 1). Eight of the 28 objects in the world were either mildly or critically injured and had to be delivered at the drop zone, whereas the other 20 were healthy.

### 3.5 Task

The objective of the task was to search and rescue the eight target victims by inspecting the different areas and dropping the correct victims on the drop zone in a specific order. During the low interdependence condition, the retrieval of the eight victims was equally divided between the autonomous virtual robot and human agent, across two separate drop zones. This way, both team members hardly depended on each other and could execute their individual actions independently. In contrast, during the high interdependence condition the eight victims had to be delivered to one shared drop zone. Consequently, the human’s actions highly depended on what the robot did (and vice versa) over a sustained sequence of actions. For both conditions, when all eight victims were rescued or when the task was not successfully completed after 10 min, the world and task were terminated and all objective data logged.

### 3.6 Agent types

We added two agents to the world: an autonomous rule-based virtual robot (called RescueBot) and a human agent controlled by the participants, using their keyboard. RescueBot was able to solve the collection task by searching for the next victim to rescue, keeping track of which areas it searched and which victims it found and where, and dropping found goal victims at the drop zone. Both agents could carry only one victim at a time, detect other agents with a range of two grid cells, detect other objects like walls and doors with an infinite sense range, and detect victims with a sense range of only one grid cell. To avoid ceiling effects resulting from a perfect agent, RescueBot moved slower than the human agent and traversed every grid cell during area exploration.

Four different versions of RescueBot were implemented for the experiment, varying with respect to what, how, and how much they communicated between communication style conditions and in capacity between the interdependence conditions. During the high interdependence condition, RescueBot lacked the capacity to carry critically injured adults and distinguish between kids, which added required/hard interdependence relationships between human and robot. When a critically injured adult was found by either RescueBot or the human participant, RescueBot told the participant to pick it up. When RescueBot found an injured kid, it told the human participant to visit that area and clarify the gender of the victim. This way, these hard dependencies required the human and robot to establish supporting interdependence relationships. It is important to emphasize that this version of RescueBot did not wrongly classify kids or unsuccessfully carry critically injured adults, but rather requested support from its human teammate.

We implemented four different communication styles for RescueBot: silent, transparent, explainable, and adaptive. The silent version served as baseline and only disclosed the crucial decisions to request human assistance in case it needed human help. In contrast, the transparent version disclosed its world knowledge/beliefs, actions, decisions, and, in the high interdependence condition, suggestions. The explainable version not only disclosed its world knowledge, actions, suggestions, and decisions, but also clarified them by providing explanations. This communication style provided attributive/causal explanations providing reasons (why) for intentional behavior and actions (Malle, 2004, 2011; Miller, 2019). The provided reasons included world knowledge, goals, and limitations and adhered to the principles of being simple (few causes), general, complete, and sound (Miller, 2019). Finally, the adaptive version of RescueBot adjusted its communication based on time pressure and relevancy. In general, after explaining a certain belief, action, suggestion, or decision *X* based on a goal, belief, or limitation reason *Y*, the agent adhered to only disclosing *X* in future situations. Moreover, when time pressure was high (less than 5 minutes remaining) RescueBot only communicated the most crucial information. The exact information content for each of the communication styles can be found in Table 1.

**TABLE 1:** The information content for each of the four communication styles. Explainability included both the content under the column Transparency, plus the explanation under the column Explanation. Underlined messages refer to the only information shared by the silent baseline. Except for the first two messages, the adaptive communication style dropped the explanations after providing them once. When time pressure was high, the adaptive version of RescueBot stopped sending the messages with the bold numbers.

Message	Transparency	Explanation
1	Moving to *X*	to pick up *Y*
2	Moving to *X*	to search for *Y*
		and because it is the closest unsearched area
**3**	Searching through whole *X*	because my sense range is limited and to find *Y*
4	Found *Y* in *X*	because you told me *Y* was located here
5	Found *Y* in *X*	because I am traversing the whole area
6	You should pick up *Y* in *X*	because I am forbidden to carry critically injured adults
7	*Y* not present in *X*	because I searched the whole area without finding *Y*
8	You should clarify the gender	
	of the injured baby in *X*	because I am unable to distinguish them
9	Going to re-search areas	to find *Y*
		and because we searched all areas but did not find *Y*
10	Picking up *Y* in *X*	because *Y* should be transported to the drop zone
**11**	Transporting *Y* to drop zone	because *Y* should be delivered there for treatment
12	Delivered *Y* at drop zone	because *Y* was current victim to rescue
13	Waiting for human at drop zone	because previous victim should be collected first
14	I suggest you pick up *Y* in *X*	because *X* is far away and you can move faster

*X* refers to specific area, *Y* to specific victim

Participants had the ability to communicate to RescueBot via the buttons shown in Figure 1B. Using these, participants could share their current and future actions, perceptions, as well as answers to any of RescueBot’s questions (in the high interdependence scenario). RescueBot added the shared information to its memory, and adjusted its behavior correspondingly. This messaging interface was present in a similar fashion as shown in Figure 1, so immediately on the right of the environment. Furthermore, the messaging interface was the same for both RescueBot and the participant, a chat box/room consisting of textual messages. Participants only had to press buttons to share required information such as which areas they searched or where they found victims. This way, we tried to decrease workload as a result of having to type messages.

RescueBot’s behavior did not vary between the four communication style conditions. When RescueBot did not know the location of the current victim to rescue, it moved towards the closest unsearched area and explored it. If the participant told RescueBot it was going to search the same area, RescueBot moved to the next closest unsearched area to explore instead. During exploration of the areas, RescueBot added the location of found victims to its memory. When participants found victims and communicated this, RescueBot also added this to its memory. If RescueBot found the current victim to rescue during area exploration, it first completed searching the whole area before picking up and dropping the victim at the drop zone. In case participants told RescueBot they would pick up the victim instead, RescueBot would start searching for or picking up the next victim to rescue. If this victim was already found by the participant, RescueBot would move to the corresponding area and explore the whole room as it did not know the exact location. When it found the victim, it would immediately pick it up and move to the drop zone rather than searching the rest of the area. During all situations described above the transparent, explainable, and adaptive versions of RescueBot communicated their actions, beliefs, decisions and suggestions using the messages outlined in Table 1.

### 3.7 Measures

#### 3.7.1 Team performance

We objectively measured team performance during the low and high interdependence conditions using completion time, accuracy, and completeness. We transformed completion time to the percentage of time left to finish the task and calculated overall team performance as the mean of time left, accuracy, and completeness. Completion time was converted into the percentage of time left in order to transform the variable to the same scale and interpretation as accuracy and completeness (i.e., expressed in % and with higher values reflecting better performance). Accuracy reflected the percentage of victims collected in the correct order, whereas completeness reflected the percentage of all victims collected. We manually kept track of accuracy during the task, while completeness was logged automatically using MATRX.

#### 3.7.2 Situation awareness of RescueBot

Situation awareness (SA) of RescueBot’s behavior processes and decisions was measured objectively, using the Situation Awareness Global Assessment Technique (SAGAT) (Endsley, 1988; Sanneman and Shah, 2020). First, we defined the human informational and SA requirements during the search and rescue task using the Goal Directed Task Analysis (Endsley, 2017). We used this analysis to define which information participants required about RescueBot’s behavior in order to achieve their respective goals. Next, we formulated eight SAGAT queries for each interdependence condition, objectively evaluating human knowledge of this situational information (Endsley, 1988, 2017). Table 2 shows all queries used during the experiment, for each interdependence condition.

**TABLE 2:** The SAGAT queries used during the experiment, for each interdependence condition.

Low Interdependence	High Interdependence
Which area(s) did RescueBot search?	Which area(s) did RescueBot search?
Which victim(s) did RescueBot find?	Which victim(s) did RescueBot find?
Where is RescueBot currently located?	Where is RescueBot currently located?
Which action is RescueBot currently executing?	Which action is RescueBot currently executing?
Which victim(s) did RescueBot find in area *Y*?	Which victim(s) did RescueBot find in area *Y*?
Which action will RescueBot perform next?	Which victim(s) did RescueBot rescue/drop?
Which of your goal victim(s) did RescueBot find?	Which victim(s) is RescueBot unable to carry?
In which area did RescueBot find victim *X*?	Which victim(s) is RescueBot unable to identify?

*X* refers to specific area, *Y* to specific victim.

During both interdependence conditions, the task was paused twice, and the same eight queries were asked. We took the average percentage of correctly answered queries as objective measure of SA. For each query we provided five multiple choice options, except for query three which was answered by selecting a location on the map. The answer options were different for each of the two assessment moments, except for queries four, six, and seven (low interdependence) because they only had five possible answer options. Finally, for queries five and eight (low interdependence) the exact area and victim used in the query was different for each assessment moment.

#### 3.7.3 Trust

We subjectively measured user trust in RescueBot using the 5-pt Likert trust scale for XAI (Hoffman et al., 2018). This scale consisted of eight items and measured confidence in and predictability, reliability, safety, efficiency, wariness, performance, and likeability of RescueBot. We calculated the mean of the eight items as the final trust score.

#### 3.7.4 Workload

Workload during the task was measured subjectively using the raw NASA Task Load Index (NASA-TLX) (Hart and Staveland, 1988). This consisted of six items evaluated on scales from 0 to 100 and increments of size five, so yielding 20 answer options. The six items measured mental, physical, and temporal demand, as well as performance, effort, and frustration. We calculated the mean of the six items as the final workload score.

#### 3.7.5 Perceived system understanding

We subjectively measured understanding of RescueBot using the 7-pt Likert Perceived System Understanding Questionnaire (van der Waa et al., 2021). This scale consisted of eight items and measured explainability, understandability, and predictability of RescueBot. We calculated the mean of the eight items as the final understanding score.

#### 3.7.6 Reliance

Reliance was objectively measured using the MATRX loggers. We defined reliance as the percentage of victims that were found first by the participant but rescued by RescueBot. Using the loggers, for each participant we counted how many of the goal victims they found first. Next, we counted how many of these victims were eventually picked up and dropped by RescueBot, and divided this by the number of victims the participant found first to get the corresponding reliance percentage.

We defined reliance in this way because it allowed the inclusion of the silent baseline into the analysis. For example, we also thought of defining reliance as the percentage of victims found by RescueBot but rescued by the participant (i.e., reliance upon RescueBot’s perceptions). However, defining reliance as such would exclude the silent baseline from the analysis, as it did not send information about RescueBot’s perceptions. Therefore, we defined reliance as the percentage of victims found by the participant but rescued by RescueBot (i.e., reliance upon RescueBot’s actions), and included the silent baseline into the analysis.

#### 3.7.7 Human rescue contribution

We measured human rescue contribution using the MATRX loggers, and defined it as the percentage of goal victims rescued by the participant.

#### 3.7.8 Human messages sent

Finally, we logged the number of messages sent from participant to RescueBot to investigate human communication frequency.

### 3.8 Procedure

The experiment was conducted in two sessions: an introduction and experiment session. The introduction session served as a tutorial aimed at getting the participants familiar with the environment, controls, and messaging system, to minimize any learning and order effects, and to control for the possible influence of gaming experience. During the tutorial, RescueBot gave the same step by step instructions to all participants, for example, on how to pick up a victim and when to send certain messages.

The second part of the tutorial included a trial of the real experiment. During this trial, the participant collaborated with the version of RescueBot with which they would also collaborate during the real experiment (so the silent, transparent, explainable, or adaptive version). Participants had to search and rescue six victims on one joint drop zone while collaborating with RescueBot, so similar to the high interdependence trial but without any required dependencies resulting from robot limitations.

After 3 minutes, we paused the trial and introduced the participants to the SAGAT queries. We explained that during the real experiment, the task would be paused at random moments and several queries would be asked related to their knowledge of RescueBot’s behavior processes and decisions. Participants were encouraged to make their best guess when they did not know or were uncertain about the answer, but we also told them that they could skip a question when they were not comfortable enough to guess.

After the tutorial, we asked the participants if they were comfortable enough to start the experiment session or wanted to re-do the trial. In the experiment session, participants completed the two interdependence conditions. Which condition the participants completed first was counterbalanced, resulting in two order conditions. We controlled for age, gender, education, and gaming experience across these two order conditions, resulting in no significant differences between the two conditions for any of these factors. During both interdependence conditions we paused the task twice, followed by the corresponding eight SAGAT queries in Table 2. The first freeze was at a random moment between two and 3 minutes after starting the task, the second freeze a minimum of one and a half minute later than the first one and a maximum of 2 minutes later.

When participants finished the first task, we presented them with the Trust Scale for XAI and NASA-TLX. Next, participants completed the second variant of the task. Again, we paused the task twice and asked the SAGAT queries. After finishing the second task, participants again completed the Trust Scale for XAI and NASA-TLX. Finally, participants filled in the Perceived System Understanding questionnaire to end the experiment. The whole study lasted for about 1 hour and was conducted during an online meeting using either Microsoft Teams, Zoom, or Google Meet. All survey responses were collected using Qualtrics.

## 4 Results

### 4.1 Learning and order effects

We examined the presence of potential learning and order effects by testing for differences in dependent variable outcomes between i) the two experiment order versions (to test for order effects) and ii) the two time points (to test for learning effects). Order 1 started with the low interdependence condition followed by the high interdependence condition, and vice versa for order 2. Time point 1 included all data from the low interdependence condition from order 1 and high interdependence condition from order 2, whereas time point 2 included all data from the low interdependence condition from order 2 and high interdependence condition from order 1.

#### 4.1.1 Order effects

We tested for order effects on all the dependent variables trust, workload, understanding, situation awareness, team performance, reliance, human rescue contribution, and human messages sent. When all assumptions were met, we conducted an independent-samples *t*-test, if not we conducted a Mann-Whitney U test. We did not find statistically significant differences in the outcome scores between the two order conditions for trust (W = 2147, *p* = 0.08), reliance (W = 2494, *p* = 0.70), workload (t (140) = 1.75, *p* = 0.08), situation awareness (W = 2412, *p* = 0.47), team performance (W = 2494, *p* = 0.70), human rescue contribution (W = 2562, *p* = 0.90), human messages sent (W = 2444, *p* = 0.55), or understanding (W = 2392, *p* = 0.43). Corresponding descriptive statistics and plots can be found in the Supplementary Materials.

#### 4.1.2 Learning effects

We tested for learning effects on the objective measures of situation awareness, team performance, reliance, and human rescue contribution to examine whether participants performed the task differently at later time points. When all assumptions were met, we conducted a paired-samples *t*-test, if not we conducted a Wilcoxon signed-rank test. We did not find statistically significant differences in the outcome scores between the two time points for situation awareness (t (71) = −0.86, *p* = 0.39), team performance (W = 1166, *p* = 0.41), human rescue contribution (W = 610, *p* = 0.12), or reliance (W = 1214, *p* = 0.25). Corresponding descriptive statistics and plots can be found in the Supplementary Materials.

### 4.2 Effects of communication style and interdependence

Here, we report the effects of and interaction between communication style and interdependence on the dependent variables. For most of the dependent variables, we employed a non-parametric rank based method for the analysis of variance (ANOVA), mainly to deal with violations of the mixed ANOVA assumption of normality. To this end, we used the *R* package and function *nparLD* (Noguchi et al., 2012) for non-parametric tests for repeated measures data in factorial designs. This method defines relative treatment effects in reference to the distributions of the dependent variables, estimated on mean ranks. Therefore, relative treatment effects can be considered as generalized expectations or means. This method does not require distributional assumptions, is applicable to a variety of data types, and is robust with respect to outliers and small sample sizes. For the dependent variables that did meet all assumptions, we conducted a mixed ANOVA.

#### 4.2.1 Trust

**FIGURE 2:**
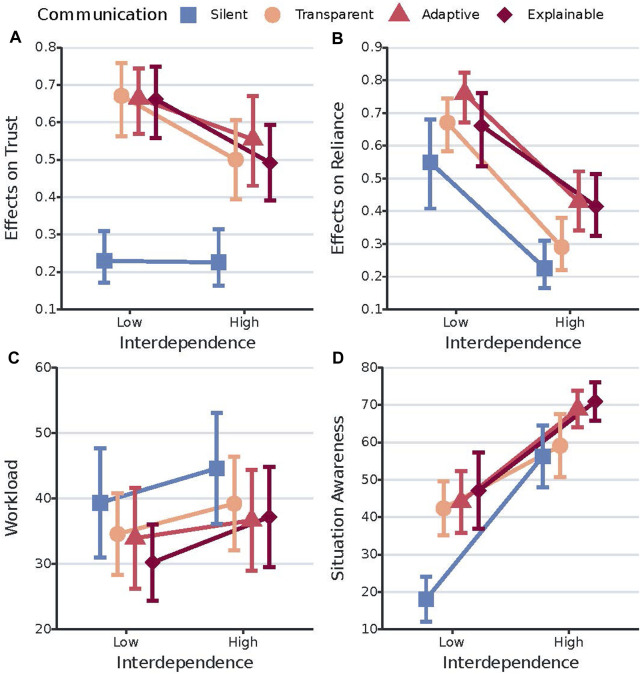
Interaction plots of the effects of communication style and interdependence on the dependent variables trust, reliance, workload, and situation awareness. **(A)** shows the relative treatment effects of communication style on trust across interdependence. The y-axis is the conventional graphical representation of the non-parametric ANOVA we used. It represents the relative marginal effect of the different communication styles across interdependence. The higher is the value on the y-axis, the higher is the corresponding trust value/score. Error bars represent the 95% confidence intervals of the relative marginal effects. **(B)** shows the relative treatment effects of communication style on reliance across interdependence. The higher is the value on the y-axis, the higher is the corresponding reliance percentage value/score. Error bars represent the 95% confidence intervals of the relative marginal effects. **(C)** shows the effects of communication style on workload across interdependence. The y-axis represents the mean workload. Error bars represent the 95% confidence intervals of the mean workload scores. **(D)** shows the effects of communication style on situation awareness across interdependence. The y-axis represents the mean situation awareness scores. Error bars represent the 95% confidence intervals of the mean situation awareness scores.

Since there were two extreme outliers and the data was not normally distributed (p < 0.05) in 2 cells of the design, as assessed by Shapiro-Wilk’s test of normality, we conducted the non-parametric rank based ANOVA. Results showed a statistically significant main effect of communication style (F (2.92) = 13.40, p < 0.0001, effect size = 0.81) on trust. Pairwise robust ATS post-hoc comparisons revealed statistically significant differences in trust scores between the silent baseline (RTE = 0.23, Mean Rank = 33.30, SD Rank = 27.63) and transparent (RTE = 0.59, Mean Rank = 84.85, SD Rank = 36.36) (F (1) = 27.39, p < 0.0001), adaptive (RTE = 0.61, Mean Rank = 88.28, SD Rank = 35.68) (F (1) = 36.43, p < 0.0001), and explainable (RTE = 0.58, Mean Rank = 83.57, SD Rank = 38.09) (F (1) = 23.24, p < 0.0001) conditions. In addition, results showed a statistically significant main effect of interdependence (F (1) = 18.76, p < 0.0001, effect size = 0.51) on trust, revealing a significant difference in trust scores between the low (RTE = 0.56, Mean Rank = 80.69, SD Rank = 41.23) and high (RTE = 0.44, Mean Rank = 64.31, SD Rank = 40.66) interdependence conditions. Figure 2A shows the interaction plot of the relative effects of communication style and interdependence on trust scores, exact relative treatment effects (RTE) and corresponding mean ranks can be found in Table 3.

**TABLE 3:** Descriptive statistics for the dependent variables trust and reliance. Values correspond to the data points of the plots in Figures 2A,B.

Variable	Communication	Interdependence	Mean Rank (SD)	RTE	95% CI
Trust	Silent	Low	33.61 (27.13)	0.23	[0.17 0.31]
	Silent	High	33.00 (28.91)	0.23	[0.16 0.31]
	Transparent	Low	97.14 (35.41)	0.67	[0.56 0.78]
	Transparent	High	72.56 (38.15)	0.50	[0.39 0.61]
	Adaptive	Low	96.19 (27.59)	0.66	[0.57 0.74]
	Adaptive	High	80.36 (41.55)	0.55	[0.43 0.67]
	Explainable	Low	95.81 (34.92)	0.66	[0.56 0.75]
	Explainable	High	71.33 (38.10)	0.49	[0.39 0.59]
Reliance	Silent	Low	79.58 (49.52)	0.55	[0.41 0.68]
	Silent	High	33.06 (27.36)	0.23	[0.17 0.31]
	Transparent	Low	97.08 (26.04)	0.67	[0.58 0.74]
	Transparent	High	42.31 (26.01)	0.29	[0.22 0.38]
	Adaptive	Low	109.83 (26.20)	0.76	[0.67 0.82]
	Adaptive	High	62.22 (30.90)	0.43	[0.34 0.52]
	Explainable	Low	95.72 (39.29)	0.66	[0.54 0.76]
	Explainable	High	60.19 (31.65)	0.41	[0.32 0.51]

#### 4.2.2 Reliance

Because the data was not normally distributed (p < 0.05) in most cells of the design, we conducted the non-parametric rank based ANOVA. Results showed a statistically significant main effect of communication style (F (2.69) = 3.99, p < 0.025, effect size = 0.38) on reliance. Pairwise robust ATS post-hoc comparisons revealed statistically significant differences in reliance scores between the silent (RTE = 0.39, Mean Rank = 56.32, SD Rank = 45.95) and explainable (RTE = 0.54, Mean Rank = 77.96, SD Rank = 39.51) condition (F (1) = 4.33, p < 0.05), silent and adaptive (RTE = 0.59, Mean Rank = 86.03, SD Rank = 37.15) condition (F (1) = 9.06, p < 0.005), and adaptive and transparent (RTE = 0.48, Mean Rank = 69.69, SD Rank = 37.81) condition (F (1) = 5.01, p < 0.05). In addition, results showed a statistically significant main effect of interdependence (F (1) = 104.30, p < 0.0001, effect size = 1.20) on reliance, revealing a statistically significant difference in reliance scores between the low (RTE = 0.66, Mean Rank = 95.56, SD Rank = 37.42) and high (RTE = 0.34, Mean Rank = 49.44, SD Rank = 31.00) interdependence conditions. Figure 2B shows the interaction plot of the relative effects of communication style and interdependence on reliance scores, exact relative treatment effects and corresponding mean ranks can be found in Table 3.

#### 4.2.3 Workload

Since all assumptions were met (no outliers, normality, homogeneity of variances, and homogeneity of covariances), we performed a mixed ANOVA. Results showed a statistically significant main effect of interdependence (F (1, 68) = 0.46, p < 0.0005, $\eta_{G}^{2}$ = 0.024) on workload. A paired-samples *t*-test was conducted to determine the effect of interdependence on workload scores. Results showed that there was a significant difference in workload scores during high (Mean = 39.40, SD = 16.7) and low (Mean = 34.50, SD = 15.40) interdependence conditions (t (71) = -3.87, p < 0.0005, d = 0.46). Figure 2C shows the interaction plot of the effects of communication style and interdependence on workload scores, Table 4 shows the descriptive statistics for each combination of communication style and interdependence.

**TABLE 4:** Descriptive statistics for the dependent variables workload and situation awareness (SA). Values correspond to the data points of the plots in Figures 2C,D.

Variable	Communication	Interdependence	Mean (SD)	95% CI
Workload	Silent	Low	39.35 (18.07)	[31.00 47.70]
	Silent	High	44.60 (18.31)	[36.14 53.06]
	Transparent	Low	34.58 (13.48)	[28.36 40.81]
	Transparent	High	39.21 (15.43)	[32.08 46.34]
	Adaptive	Low	33.93 (16.68)	[26.23 41.64]
	Adaptive	High	36.67 (16.59)	[29.00 44.33]
	Explainable	Low	30.23 (12.59)	[24.41 36.04]
	Explainable	High	37.18 (16.52)	[29.54 44.81]
SA	Silent	Low	18.06 (13.02)	[12.04 24.07]
	Silent	High	56.25 (17.83)	[48.01 64.48]
	Transparent	Low	42.36 (15.69)	[35.11 49.61]
	Transparent	High	59.12 (18.30)	[50.67 67.58]
	Adaptive	Low	44.10 (17.87)	[35.84 52.35]
	Adaptive	High	68.87 (10.55)	[64.00 73.75]
	Explainable	Low	47.12 (22.03)	[36.95 57.30]
	Explainable	High	70.93 (11.13)	[65.79 76.07]

#### 4.2.4 Situation awareness

Because all assumptions were met we conducted a mixed ANOVA. Results showed a statistically significant interaction between communication style and interdependence on situation awareness (SA) scores (F (3, 68) = 3.31, p < 0.05, $\eta_{G}^{2}$ = 0.057). We analyzed the simple main effect of communication on SA during each interdependence condition using a one-way ANOVA. Results showed that the simple main effect of communication style was significant during both the high (F (3, 68) = 4.20, p < 0.025, $\eta_{G}^{2}$ = 0.156) and low (F (3, 68) = 10.60, p < 0.0001, $\eta_{G}^{2}$ = 0.318) interdependence conditions. Pairwise comparisons using a Bonferroni correction revealed significant differences in SA scores between the silent baseline (Mean = 18.06, SD = 13.02) and transparent (Mean = 42.36, SD = 15.69, p < 0.001), adaptive (Mean = 44.10, SD = 17.87, p < 0.0005), and explainable (Mean = 47.12, SD = 22.03, p < 0.0001) conditions when interdependence was low. When interdependence was high, results showed a significant difference in SA scores between the silent baseline (Mean = 56.25, SD = 17.83) and only the explainable condition (Mean = 70.93, SD = 11.13, p < 0.05).

We analyzed the simple main effect of interdependence on SA for each communication condition using a paired-samples *t*-test. Results showed statistically significant differences in mean SA scores between the high and low interdependence conditions for the silent (Mean High = 56.20, SD High = 17.80; Mean Low = 18.10, SD Low = 13.00) (t (17) = -10.00, p < 0.0001, d = 2.36), transparent (Mean High = 59.10, SD High = 18.30; Mean Low = 42.40, SD Low = 15.70) (t (17) = -3.07, p < 0.01, d = 0.72), adaptive (Mean High = 68.90, SD High = 10.60; Mean Low = 44.10, SD Low = 17.90) (t (17) = -4.68, p < 0.0005, d = 1.10), and explainable (Mean High = 70.90, SD High = 11.1; Mean Low = 47.10, SD Low = 22.00) (t (17) = -4.84, p < 0.0005, d = 1.14) conditions. Figure 2D shows the interaction plot of the effects of communication style and interdependence on SA scores, Table 4 shows the descriptive statistics for each combination of communication style and interdependence.

#### 4.2.5 Team performance

**FIGURE 3:**
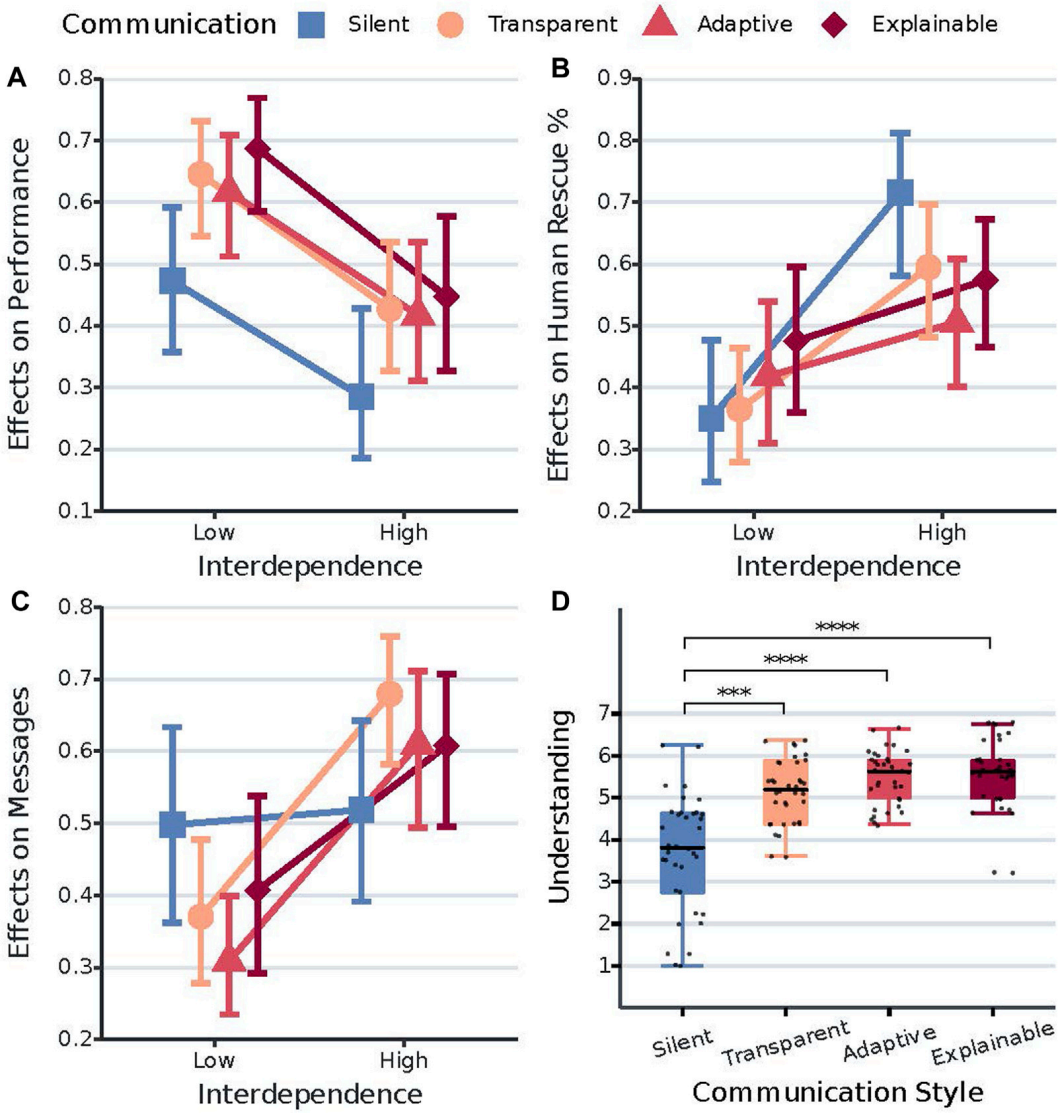
Interaction plots of the effects of communication style and interdependence on the dependent variables team performance, human rescue contribution, and human messages sent **(A,B,C)**. Boxplots of system understanding for each of the communication style conditions **(D)**. **(A)** shows the relative treatment effects of communication style on performance across interdependence. The y-axis is the conventional graphical representation of the non-parametric ANOVA we used. It represents the relative marginal effect of the different communication styles across interdependence. The higher the value on the y-axis, the higher is the corresponding performance value/score. Error bars represent the 95% confidence intervals of the relative marginal effects. **(B)** shows the relative treatment effect of communication style on human rescue contribution across interdependence. The higher the value on the y-axis, the higher is the corresponding human rescue percentage value/score. Error bars represent the 95% confidence intervals of the relative marginal effects. **(C)** shows the relative treatment effects of communication style on human messages sent across interdependence. The higher the value on the y-axis, the higher is the corresponding number of messages sent by the participants. Error bars represent the 95% confidence intervals of the relative marginal effects. **(D)** shows the effects of communication style on system understanding. The y-axis represents the mean understanding scores. ***p < 0.0005. ****p < 0.0001.

Since the data was not normally distributed in most cells of the experimental design, we conducted the non-parametric rank based ANOVA. Results showed a statistically significant main effect of interdependence (F (1) = 76.81, p < 0.0001, effect size = 1.03) on performance, revealing a statistically significant difference in team performance between the low (RTE = 0.61, Mean Rank = 87.70, SD Rank = 36.54) and high (RTE = 0.39, Mean Rank = 57.30, SD Rank = 41.23) interdependence conditions. Figure 3A shows the interaction plot of the relative effects of communication style and interdependence on performance scores, exact relative treatment effects (RTE) and corresponding mean ranks can be found in Table 5.

**TABLE 5:** Descriptive statistics for the dependent variables team performance, human rescue contribution, and number of human messages sent. Values correspond to the data points of the plots in Figures 3A–C.

Variable	Communication	Interdependence	Mean Rank (SD)	RTE	95% CI
Performance	Silent	Low	68.58 (43.65)	0.47	[0.36 0.59]
	Silent	High	41.64 (44.40)	0.29	[0.19 0.43]
	Transparent	Low	93.47 (30.80)	0.65	[0.54 0.73]
	Transparent	High	62.03 (36.31)	0.43	[0.33 0.54]
	Adaptive	Low	89.31 (32.35)	0.62	[0.51 0.71]
	Adaptive	High	60.61 (38.10)	0.42	[0.31 0.54]
	Explainable	Low	99.44 (33.23)	0.69	[0.59 0.77]
	Explainable	High	64.92 (44.73)	0.44	[0.33 0.58]
Contribution	Silent	Low	50.97 (40.83)	0.35	[0.25 0.48]
	Silent	High	103.56 (40.31)	0.72	[0.58 0.81]
	Transparent	Low	53.06 (30.73)	0.37	[0.28 0.46]
	Transparent	High	86.19 (37.23)	0.60	[0.48 0.70]
	Adaptive	Low	60.78 (40.97)	0.42	[0.31 0.54]
	Adaptive	High	73.33 (36.35)	0.51	[0.40 0.61]
	Explainable	Low	68.97 (40.27)	0.48	[0.36 0.60]
	Explainable	High	83.14 (34.57)	0.57	[0.47 0.67]
Human Messages	Silent	Low	72.19 (51.81)	0.50	[0.36 0.63]
	Silent	High	75.19 (48.71)	0.52	[0.39 0.64]
	Transparent	Low	53.83 (30.60)	0.37	[0.28 0.48]
	Transparent	High	98.33 (27.62)	0.68	[0.58 0.76]
	Adaptive	Low	44.97 (25.57)	0.31	[0.23 0.40]
	Adaptive	High	88.33 (36.11)	0.61	[0.49 0.71]
	Explainable	Low	59.11 (43.41)	0.41	[0.29 0.54]
	Explainable	High	88.03 (36.95)	0.61	[0.49 0.71]

#### 4.2.6 Human rescue contribution

Because the data was not normally distributed in most cells, we conducted the non-parametric rank based ANOVA. Results showed a statistically significant interaction between communication style and interdependence on human rescue contribution (F (2.95) = 3.03, p < 0.05, effect size = 0.35). We analyzed the simple main effect of communication on human rescue contribution during each interdependence condition using a Kruskal-Wallis test. Results showed that the simple main effect of communication style was not significant during both interdependence conditions. We analyzed the simple main effect of interdependence on human rescue contribution for each communication condition using the relative treatment effects test. Results showed a statistically significant difference in relative treatment effects between the low and high interdependence conditions for the silent (RTE Low = 0.35, RTE High = 0.72, p < 0.0001) and transparent (RTE Low = 0.37, RTE High = 0.60, p < 0.005) conditions. Figure 3B shows the interaction plot of the relative effects of communication style and interdependence on human rescue contribution scores, exact relative treatment effects (RTE) and corresponding mean ranks can be found in Table 5.

#### 4.2.7 Number of human messages sent

Since most assumptions of a mixed ANOVA were violated, we conducted the non-parametric rank based ANOVA. Results showed a statistically significant interaction between communication style and interdependence on the number of human messages sent (F (2.77) = 5.45, p < 0.005, effect size = 0.57). We analyzed the simple main effect of robot communication style on human messages sent using a Kruskal-Wallis test. Results showed that the simple main effect of communication style was not significant during both interdependence conditions. Next, we analyzed the simple main effect of interdependence on human messages sent for each communication style condition using the relative treatment effects test. Results showed a statistically significant difference in relative treatment effects between the low and high interdependence conditions for the transparent (RTE Low = 0.37, RTE High = 0.68, p < 0.0001), adaptive (RTE Low = 0.31, RTE high = 61, p < 0.0001), and explainable (RTE Low = 0.41, RTE High = 0.61, p < 0.01, r = 0.56) conditions. Figure 3C shows the interaction plot of the relative effects of communication style and interdependence on human messages sent, exact relative treatment effects (RTE) and corresponding mean ranks can be found in Table 5.

#### 4.2.8 System understanding

Because the data was not normally distributed in 1 cell of the design and since there was no homogeneity of variances, we conducted a Kruskal-Wallis test. Results showed that there were statistically significant differences in system understanding between the communication style conditions (*χ* ^2^ (3) = 48.30, p < 0.0001, *η* ^2^ = 0.32). Pairwise comparisons using Dunn’s procedure with a Bonferonni correction revealed statistically significant differences in understanding scores between the silent baseline (Mean Rank = 32.72, SD Rank = 31.51) and transparent (Mean Rank = 73.83, SD Rank = 36.93) (p < 0.0005), adaptive (Mean Rank = 89.72, SD Rank = 32.74) (p < 0.0001), and explainable (Mean Rank = 93.72, SD Rank = 35.59) (p < 0.0001) conditions. Figure 3D shows the boxplots of system understanding for each of the communication style conditions.

### 4.3 Predicting team performance

We ran a multiple linear regression analysis to determine whether we could predict a quantitative outcome of team performance based on the predictor variables situation awareness, trust, reliance, workload, and human messages sent. Moreover, we added experiment version as a predictor to examine the presence of potential order effects and interdependence as an interaction term to examine whether the association between predictors and outcome depended on the level of interdependence. Next, we used the GVLMA package to check the linear model assumptions normality, heteroscedasticity, linearity, and uncorrelatedness of the model. Since not all assumptions were acceptable, we removed the five unusual observations (out of a total of 144 observations).

**FIGURE 4:**
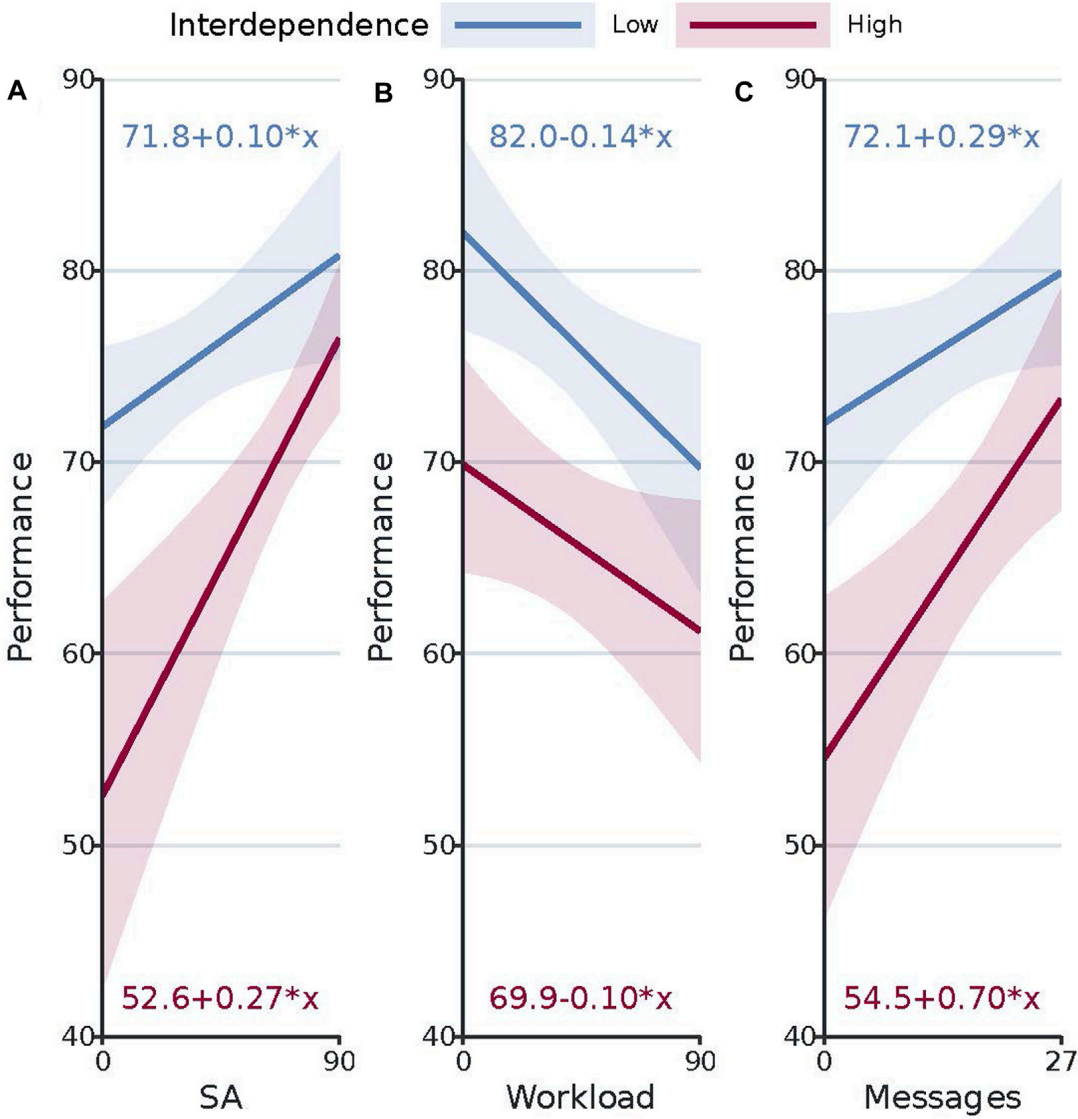
Predicted values of team performance based on the statistically significant predictor variables situation awareness (SA), workload, and human messages sent. Intervals represent the lower and upper bounds of the 95% confidence intervals for the predicted values. **(A)** shows the predicted changes in team performance with changes in the predictor variable situation awareness, at both interdependence levels. **(B)** shows the predicted changes in team performance with changes in the predictor variable workload, at both interdependence levels. **(C)** shows the predicted changes in team performance with changes in the predictor variable human messages sent, at both interdependence levels.

Results showed that the regression model statistically significantly predicted team performance (F (13, 125) = 7.11, p < 0.0001, adj. R^2^ = 0.37). Furthermore, results showed that only situation awareness (p < 0.0001), workload (p < 0.005), and human messages sent (p < 0.001) added statistically significantly to the prediction. When interdependence was low an increase in SA of 1% was associated with an increase in team performance of 0.10%, an increase in workload of 1% with a decrease in team performance of 0.14%, and an increase in human messages sent of 1 message with an increase in team performance of 0.29%. When interdependence was high an increase in SA of 1% was associated with an increase in team performance of 0.27%, an increase in workload of 1% with a decrease in team performance of 0.10%, and an increase in human messages sent of 1 message with an increase in team performance of 0.70%. Results also showed that the association between situation awareness and team performance depended on the level of interdependence (p < 0.05), with team performance increasing at a higher rate with an increase in SA when interdependence was high. Figure 4 shows the interaction plots of the significant predictors. Finally, experiment version (i.e., order) did not add statistically significantly to the prediction of team performance.

## 5 Discussion and conclusion

### 5.1 Discussion

#### 5.1.1 Trust

The results in Section 4.2.1, Figure 2A, and Table 3 clearly show that robot communication results in significantly higher trust in the robot. However, we did not find evidence for higher trust in the robot when being explainable rather than transparent. This is not in line with other studies demonstrating how providing more reasoning information is related to increases in trust (Boyce et al. (2015); O’Neill et al. (2020). Furthermore, we observe that trust in the robot significantly decreases when interdependence is high, which does not correspond with other studies reporting that increasing interdependence subsequently increases participant positive affect (Walliser et al., 2017, 2019; O’Neill et al., 2020). One possible explanation is that we increased interdependence not only by removing the work division, but also by adding hard interdependence relationships stemming from a lack of robot capacity. Therefore, we believe it is crucial to carefully consider the details of task interdependence and interdependence relationships when comparing studies on interdependence in human-robot teams. For example, two human-robot teams can be highly interdependent but if one team is characterized by hard interdependencies stemming from a lack of robot capacity and the other by hard interdependencies stemming from a lack of human capacity, it is not surprising when trust in the robot differs significantly between these two teams. Another possible reason for this result is that trust is only critical when human and robot are highly interdependent, and therefore people judge it more critically. This is in line with the claim that interdependence relationships are the mechanisms by which relational trust is established (Johnson and Bradshaw, 2021).

#### 5.1.2 Reliance

For reliance the results show that people rely more on RescueBot when it provides explanations, as demonstrated by the significant difference in reliance scores between the explainable and silent condition, and adaptive and both silent and transparent conditions. This could be the result of the specific message/explanation number one from Table 2, explaining the reason why RescueBot was moving to a certain area (to pick up a certain victim). It is possible that when people received this explanation, they were more inclined to let RescueBot complete this goal rather than engage in the same tasks, showing benefits to the coordination of work. The results further show a significant decrease in reliance during high interdependence, as clearly visualized in Figure 2B. This could be explained by the similar significant decrease in trust scores when interdependence is high. Since trust can impact reliance upon and use of autonomous AI systems (Parasuraman and Riley, 1997; Dzindolet et al., 2003; Schaefer et al., 2017), the decrease in trust during high interdependence might have resulted in a corresponding decrease in reliance upon RescueBot to rescue victims found by the human participants.

#### 5.1.3 Workload

Results further show no evidence for an increase in workload when adding explanations to transparency, which is in line with the results in Mercado et al. (2016); Chen et al. (2018); Selkowitz et al. (2017). This indicates that dynamic adaptation based on workload might not be necessary in this type of task and scenario. Furthermore, we observe a higher workload when human and robot are highly interdependent. This could be due to the increasing importance of coordination and collaboration (Johnson et al., 2014; Singh et al., 2017), resulting in more human effort to stay aware of the robot’s behavior and inform the robot about own behaviorThis might be the result of the high interdependence condition having a more difficult task, which corresponds with earlier work showing how task difficulty is associated with higher workload (Wright and Kaber, 2005; Fan et al., 2010; O’Neill et al., 2020). Another possibility is that being highly interdependent requires more human effort, because it is crucial to stay aware of the robot’s behavior.

#### 5.1.4 Situation awareness

The results also show how the effects of communication style on situation awareness depend on the level of interdependence. More specifically, robot communication results in a significantly higher situation awareness when interdependence is low. However, when interdependence is high only repeatedly providing explanations results in a significantly higher situation awareness than being silent. One possible explanation for these results is that during the high interdependence scenario, the silent baseline did communicate something: the information related to the required dependencies (asking for help). However, it seems unlikely that sharing only this information can account fully for this finding. Therefore, another possibility is that being highly interdependent increases SA irrespective of communication style, as it is more necessary to complete the task well and people, therefore, pay more attention already. This is also underlined by the significantly higher SA during high interdependence, observed for all communication style conditions. As people seem to already pay more or less attention to their robot teammate depending on interdependence levels, it is crucial to understand these levels when designing explanations for any type of future applications. A final suggestion is that only explainability adds crucial information required for a higher situation awareness than just being highly interdependent can already account for.

#### 5.1.5 Team performance

For team performance, we first observe a significant decrease in performance scores when interdependence is high. This does not align with other studies reporting how increasing interdependence subsequently increases team performance (Walliser et al., 2017, 2019; O’Neill et al., 2020). One possible explanation is that increasing interdependence also increases the need for coordination and collaboration (Johnson et al., 2014; Singh et al., 2017), which in turn results in a more challenging and demanding scenario. This interpretation also aligns with the observed increase in communication frequency (for all conditions except the baseline) and higher workload when interdependence is high. Consequently, this more challenging and demanding scenario could have resulted in a decrease in performance.

The results further show that in our task and scenario, only situation awareness, workload, and human messages sent are significantly associated with team performance. More specifically, increasing SA, decreasing workload, and increasing the number of human messages sent are associated with increases in team performance. In terms of human messages sent, the result can be considered surprising as earlier work showed that a greater number of team messages shared was associated with lower team performance (Cooke et al., 2016). It is also surprising that our results do not show evidence for a significant positive association between trust and team performance, as previous works in both human-human and human-robot teams did (Korsgaard et al., 1995; Zaheer et al., 1998; Wieselquist et al., 1999; You and Robert, 2018). This suggests that (in our task and scenario) situation awareness, workload, and human messages sent are more important to team performance than trust in the robot. However, when the task and scenario get more risky, trust in the robot may become more important (Johnson and Bradshaw, 2021).

We also observe how increasing SA is associated with a significantly higher increase in team performance when interdependence is high. All in all, our results provide valuable insights into the mechanisms driving HART performance (O’Neill et al., 2020). Based on these results, we advice to pay special attention to SA, workload, and communication when designing/developing human-robot teams, while also accounting for the level of interdependence between human and robot.

#### 5.1.6 Human rescue contribution

Results further show how the effect of interdependence on human rescue contribution depends on the communication style of RescueBot. More specifically, we observe a significant increase in contribution during high interdependence only when collaborating with the silent and transparent versions of RescueBot. Put differently, only participants who did not receive explanations for robot behavior significantly increased contribution during high interdependence. This suggests that interdependence only increases human contribution when the robot does not provide explanations for its behavior. Therefore, we speculate explanations are important to the coordination of work between team members and can diminish the effect of interdependence on contribution.

#### 5.1.7 Number of human messages sent

Another result of note is the interaction between robot communication style and interdependence on the number of human messages sent. Results show how people increase their amount of messages during high interdependence only when RescueBot also communicates. This suggests that only people collaborating with the communicating robots adjust their communication frequency according to interdependence, highlighting a surprising effect of robot communication on human communication.

#### 5.1.8 System understanding

Finally, we observe significantly higher understanding scores when RescueBot communicates. However, our results provide no evidence that adding explanations to transparency results in significantly higher understanding. This is not in line with other studies demonstrating how providing more reasoning information is related to increases in perceived understanding of the autonomous agent (Harbers et al., 2011; O’Neill et al., 2020).

### 5.2 Limitations and future work

#### 5.2.1 Limitations

We identify a few limitations of our study. First, we chose to conduct the experiment in an online setting as this allowed us to simplify the task, remove robot-specific capabilities from the considerations, and keep a safe distance from participants during the global pandemic. This does mean that we did not have physical embodiment for our agent, as a robot would have. This embodiment might influence how much attention people pay to the agent when it is in sight (Looije et al., 2012). On the other hand, as many tasks (including search and rescue) would always incorporate virtual messages due to distance between team mates, we do not expect it to change our main findings.

Furthermore, our simplified and simulated environment raises questions about the ecological validity. While this environment made it relatively easy to program our agents, environment, task, and communication protocols, it is hard to determine how well these generalize to real world scenarios. Currently, state of the art urban search and rescue robots or drones are not approaching the levels of autonomy and communication presented in our work. However, we believe that the rapid developments in the fields of Robotics and Artificial Intelligence will definitely allow these levels of autonomy and communication to be achieved.

Another limitation concerns the use of only attributive/causal explanations providing reasons for intentional behavior and actions of RescueBot. The absence of large differences between the transparent, explainable, and adaptive conditions could be the result of these explanations not adding enough additional information in our task and scenario. However, we used this explanation type as they could consistently be provided with each message sent by RescueBot without increasing message length dramatically. For example, confidence, contrastive, and counterfactual explanations could not be provided with each message (especially confidence explanations) or would increase message length considerably (contrastive and counterfactual explanations specifically).

Finally, our mixed design introduced some potential confounds such as learning and order effects. We actively tried to address these potential effects by including an extensive tutorial before participants started with the real experiment. This way, we tried to ensure all participants had similar and decent entry levels before starting the real experiment. We still tested for potential order effects by i) testing for differences in dependent variable outcomes between the two experiment order versions and ii) including interdependence condition order as a predictor in our regression model predicting team performance. Both analyses did not provide evidence for the presence of such order effects, as we did not find significant differences in outcome scores between the order conditions and order did not add significantly to the prediction of team performance (see Sections 4.1, 4.3). Furthermore, we also tested for potential learning effects by testing for differences in the dependent variable outcomes between the two time points. Again, our analysis did not provide evidence for the presence of such effects (see Section 4.1), suggesting our tutorial worked as intended and our mixed experimental design did not introduce learning or order effects.

#### 5.2.2 Future work

We identify several possible directions for future work. We did not find large effects of adapting the message content, but this might still have an effect if done in other ways. Particularly, personalization by tailoring communication using an explicit user model and based on factors like user workload, trust in the agent, or understanding of the system. For example, the system could model the human using observations and human communication and adjust its information sharing accordingly. Other interesting contextual factors to investigate in future work include adapting information sharing based on different team member roles (e.g., supervisor vs. assistant) or team tasks. Another suggestion for future work is to add different explanation types such as confidence, contrastive, and counterfactual explanations and investigate their importance during human-robot teamwork on metrics such as trust and understanding.

In future work it could also be interesting to add bidirectional required dependencies to the human-robot team. In our current task and scenario, only RescueBot lacked capacity resulting in required dependencies. However, in future work, required dependencies stemming from a lack of human capacity could also be added, resulting in required support from RescueBot (e.g., with removing obstacles). Furthermore, soft interdependence relationships could be added, for example when carrying a victim together would be faster than carrying alone. It would be interesting to examine how these scenarios affect trust in the system compared to our scenario of unidirectional required dependencies. As a final suggestion for future work, it could be relevant to look into more complex and realistic scenarios and environments which more closely resemble the current state of the art search and rescue robots.

### 5.3 Conclusion

Our study shows that the distinguished styles of robot communication result in more trust in and understanding of the robot, without increasing workload during the task. This highlights the fundamental importance of robots communicating their behavior to human teammates during teamwork. Furthermore, our findings show that robot explanations result in more reliance upon that robot, and that compared to sharing nothing, only explainability results in a higher situation awareness when interdependence is high. This highlights how robots providing explanations for their behavior can benefit human-robot teamwork. Finally, results demonstrate that being highly interdependent decreases trust, reliance, and team performance while increasing workload and situation awareness. It also increases human communication frequency when the robot communicates to its human teammate, human rescue contribution when the robot does not provide explanations, and the strength of the positive association between situation awareness and team performance. This underlines the crucial importance of carefully considering interdependence during studies on human-robot teamwork.

Overall, our results show that there are important differences between being transparent, explainable or adaptive in communications, but that the level of interdependence between human and robot is crucial in determining the exact effect that communication style has on human-robot teamwork. Our findings highlight the importance of interdependence on studies into explainability in robots, and provide an important first step in determining how a robot should communicate to its human teammates.

## Data Availability

The datasets presented in this study can be found in online repositories. The names of the repository/repositories and accession number(s) can be found below: Data underlying the research project: Interdependence and Communication Style in Human-Agent/Robot Teamwork–https://doi.org/10.4121/20216708; Software and code for the research project: Interdependence and Communication Style in Human-Agent/Robot Teamwork–https://doi.org/10.4121/20216720.
