# Supplementary material for: The influence of interdependence and a transparent or explainable communication style on human-robot teamwork
Source: Front Robot AI. 2022 Sep 8;9:993997. doi: 10.3389/frobt.2022.993997 (PMC9493028; doi:10.3389/frobt.2022.993997)
Supplement: Supplementary file 1 [file DataSheet1.PDF]

Supplementary Material

1 SUPPLEMENTARY TABLES AND FIGURES

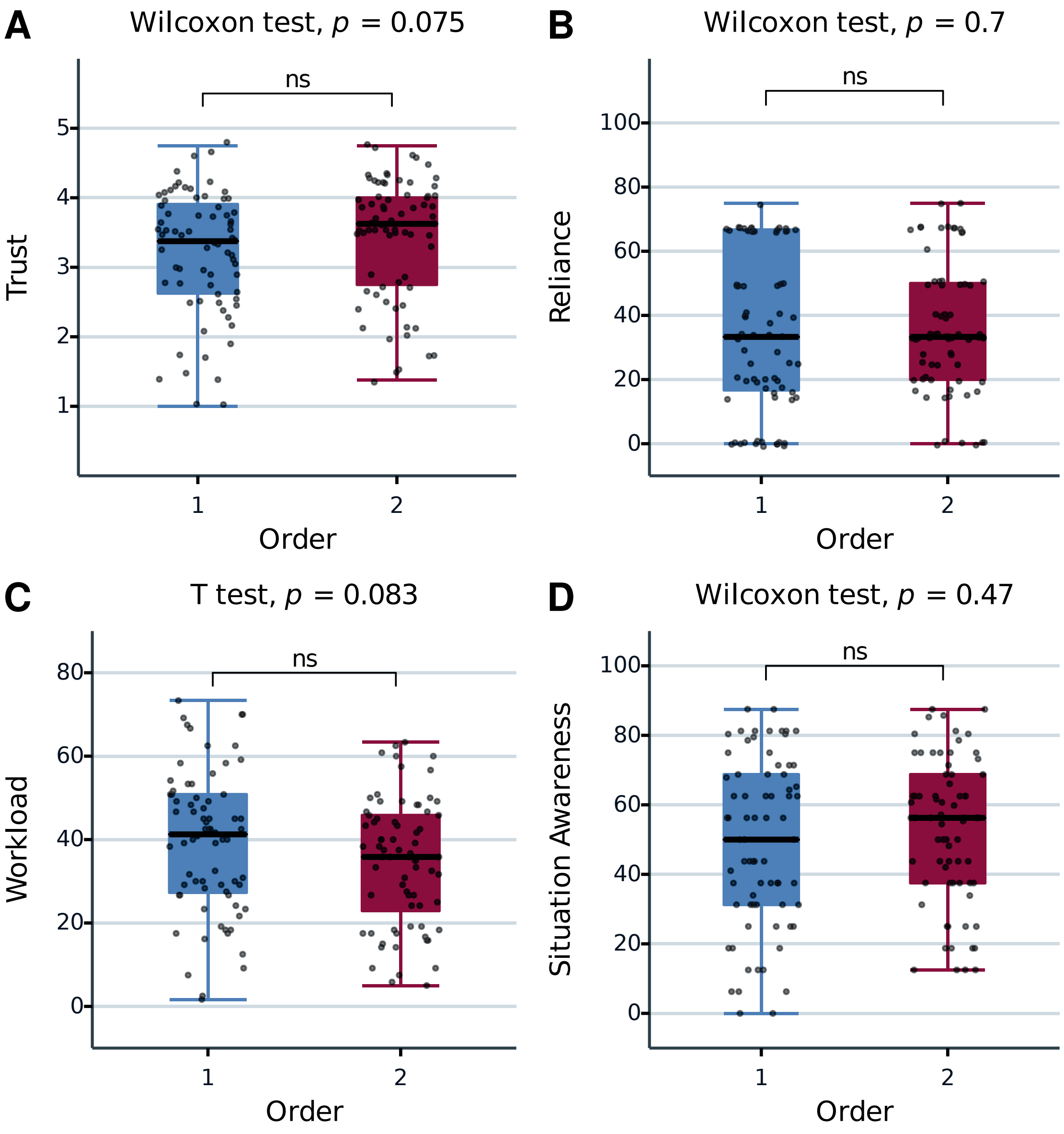

Figure S1. Boxplots of trust (**A**), reliance (**B**), workload (**C**), and situation awareness (**D**) for both order conditions. Order 1 started with the low interdependence condition followed by the high interdependence condition. Order 2 started with the high interdependence condition followed by the low interdependence condition.

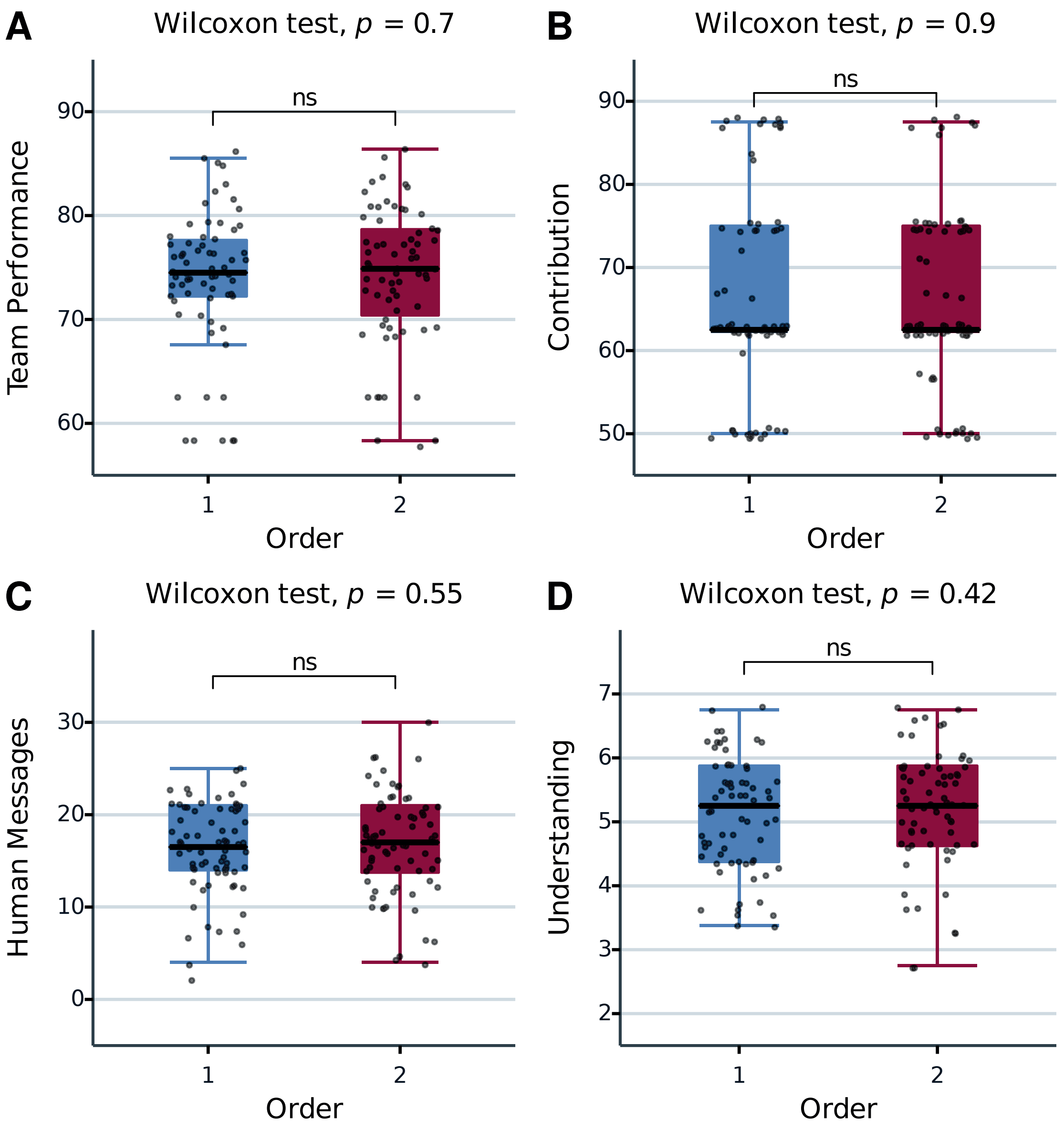

Figure S2. Boxplots of team performance (**A**), human rescue contribution (**B**), human messages sent (**C**), and system understanding (**D**) for both order conditions. Order 1 started with the low interdependence condition followed by the high interdependence condition. Order 2 started with the high interdependence condition followed by the low interdependence condition.

Variable	Order	Mean (SD)	Mean Rank (SD)	Median (IQR)
Trust	1	3.21 (0.89)	66.32 (40.20)	3.38 (1.28)
	2	3.44 (0.87)	78.68 (42.37)	3.62 (1.25)
Reliance	1	34.60 (24.40)	71.15 (45.12)	33.30 (50.00)
	2	35.70 (29.90)	73.85 (37.43)	33.30 (30.00)
Workload	1	39.30 (17.00)	78.53 (42.93)	41.20 (23.50)
	2	34.60 (15.00)	66.47 (39.82)	35.80 (22.90)
SA	1	49.00 (24.20)	70.01 (44.26)	50.00 (37.50)
	2	52.70 (20.60)	74.99 (39.01)	56.20 (31.20)
Performance	1	71.10 (12.20)	71.15 (40.77)	74.10 (7.03)
	2	72.00 (10.80)	73.85 (42.87)	74.40 (9.27)
Contribution	1	67.30 (15.30)	72.08 (43.65)	62.50 (13.10)
	2	67.10 (12.00)	72.92 (37.48)	62.50 (12.50)
Messages	1	16.20 (4.95)	70.44 (40.00)	16.50 (7.00)
	2	16.80 (5.50)	74.56 (43.33)	17.00 (7.25)
Understanding	1	4.92 (1.19)	69.72 (42.21)	5.06 (1.31)
	2	5.02 (1.22)	75.28 (41.22)	5.25 (1.16)

Table S1. Descriptive statistics for trust, reliance, workload, situation awareness, team performance, human rescue contribution, human messages sent, and understanding for both order conditions. Order 1 started with the low interdependence condition followed by the high interdependence condition. Order 2 started with the high interdependence condition followed by the low interdependence condition.

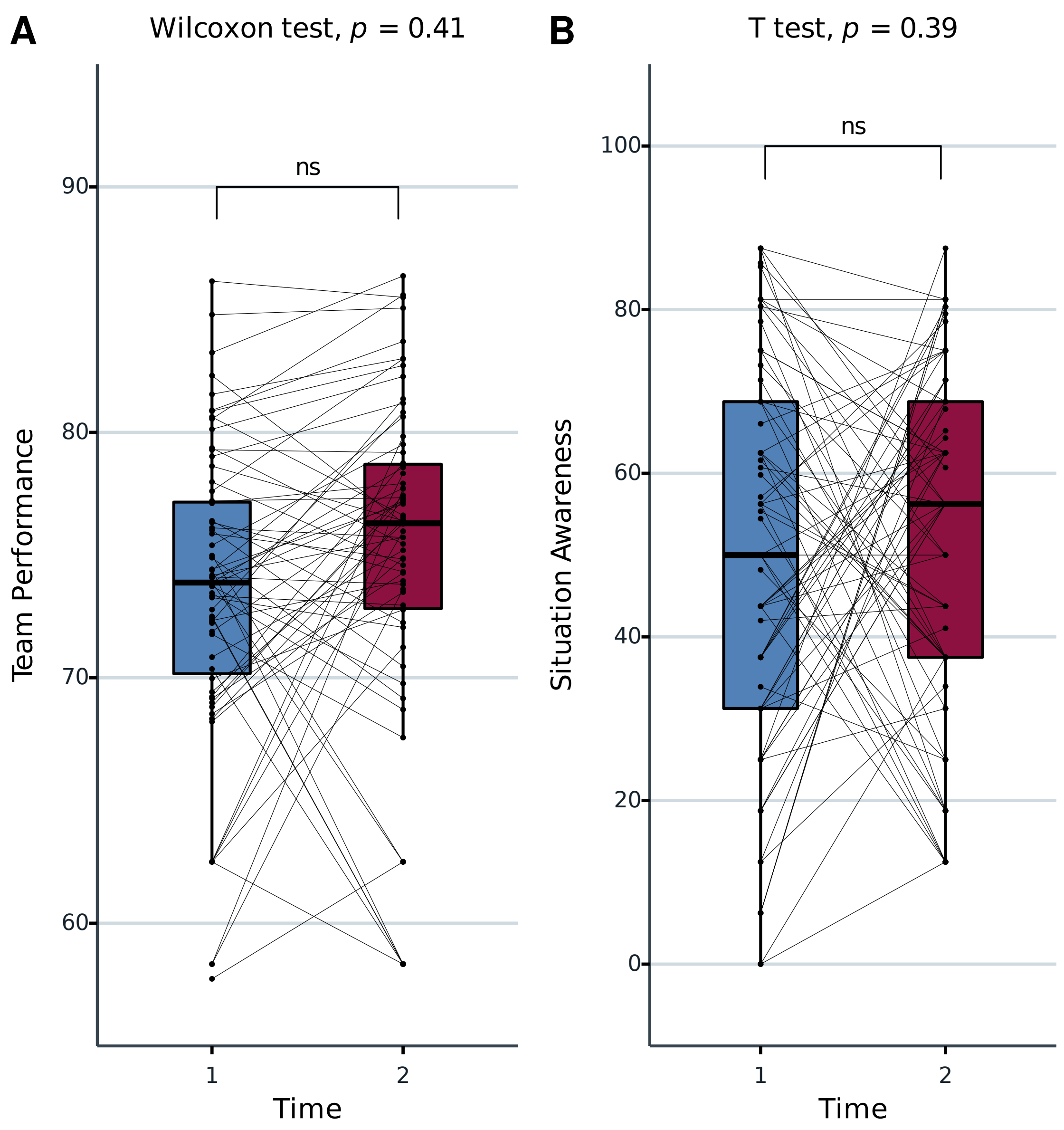

Figure S3. Boxplots of team performance (**A**) and situation awareness (**B**) at each time point. Time point 1 includes all data from the low interdependence condition from order 1 and high interdependence condition from order 2. Time point 2 includes all data from the low interdependence condition from order 2 and high interdependence condition from order 1.

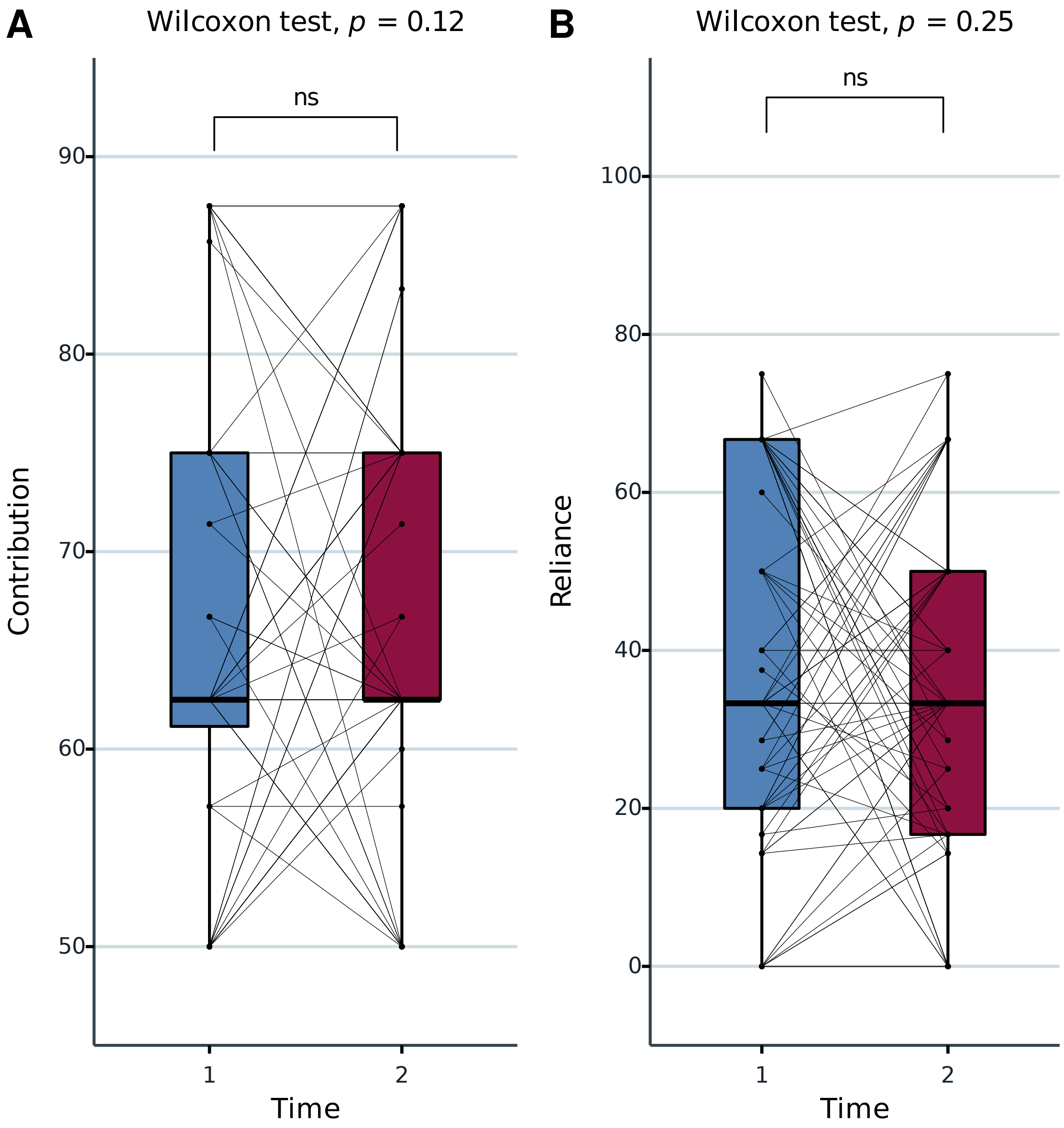

Figure S4. Boxplots of human rescue contribution (**A**) and reliance (**B**) at each time point. Time point 1 includes all data from the low interdependence condition from order 1 and high interdependence condition from order 2. Time point 2 includes all data from the low interdependence condition from order 2 and high interdependence condition from order 1.

Variable	Time	Mean (SD)	Mean Rank (SD)	Median (IQR)
Performance	1	71.60 (8.91)	66.78 (39.32)	73.60 (7.46)
	2	71.50 (13.60)	78.22 (43.49)	75.70 (8.10)
SA	1	49.10 (23.90)	69.44 (43.34)	50.00 (37.50)
	2	52.60 (21.10)	75.56 (39.94)	56.20 (31.20)
Contribution	1	65.40 (13.50)	67.08 (40.00)	62.50 (17.90)
	2	69.00 (13.80)	77.92 (40.63)	62.50 (12.50)
Reliance	1	37.40 (23.00)	76.31 (42.38)	33.30 (46.70)
	2	32.90 (21.20)	68.69 (40.18)	33.30 (33.30)

Table S2. Descriptive statistics for team performance, situation awareness, human rescue contribution, and reliance for both time points.
